# Diffusion Retardation by Binding of Tobramycin in an Alginate Biofilm Model

**DOI:** 10.1371/journal.pone.0153616

**Published:** 2016-04-21

**Authors:** Bao Cao, Lars Christophersen, Mette Kolpen, Peter Østrup Jensen, Kim Sneppen, Niels Høiby, Claus Moser, Thomas Sams

**Affiliations:** 1 Department of Clinical Microbiology, Copenhagen University Hospital, Rigshospitalet, DK-2100 Copenhagen, Denmark; 2 Department of Immunology and Microbiology, UC-CARE, Faculty of Health Sciences, University of Copenhagen, DK-2200 Copenhagen, Denmark; 3 Niels Bohr Institute, University of Copenhagen, Blegdamsvej 17, DK-2100 Copenhagen, Denmark; 4 Biomedical Engineering, Dept. of Electrical Engineering, Technical University of Denmark, DK-2800 Lyngby, Denmark; Ghent University, BELGIUM

## Abstract

Microbial cells embedded in a self-produced extracellular biofilm matrix cause chronic infections, e. g. by *Pseudomonas aeruginosa* in the lungs of cystic fibrosis patients. The antibiotic killing of bacteria in biofilms is generally known to be reduced by 100–1000 times relative to planktonic bacteria. This makes such infections difficult to treat. We have therefore proposed that biofilms can be regarded as an independent compartment with distinct pharmacokinetics. To elucidate this pharmacokinetics we have measured the penetration of the tobramycin into seaweed alginate beads which serve as a model of the extracellular polysaccharide matrix in *P. aeruginosa* biofilm. We find that, rather than a normal first order saturation curve, the concentration of tobramycin in the alginate beads follows a power-law as a function of the external concentration. Further, the tobramycin is observed to be uniformly distributed throughout the volume of the alginate bead. The power-law appears to be a consequence of binding to a multitude of different binding sites. In a diffusion model these results are shown to produce pronounced retardation of the penetration of tobramycin into the biofilm. This filtering of the free tobramycin concentration inside biofilm beads is expected to aid in augmenting the survival probability of bacteria residing in the biofilm.

## Introduction

Aggregates of microbial cells embedded in a self-produced extracellular matrix, otherwise known as biofilm, lead to chronic and recurrent infections, e. g. by *Pseudomonas aeruginosa* bacteria in the lungs of cystic fibrosis (CF) patients, which are difficult to treat [1]. In CF patients, antibiotics like tobramycin are given both intravenously and topically in the lungs by inhalation. The biofilm mode of growth provides protection of the microbial cells making them significantly less susceptible to antimicrobial treatment compared to their planktonic counterparts [2]. This feature has been attributed to a number of mechanisms such as oxygen gradients within the biofilm resulting in zones with minimal or no growth, adaptive stress responses, and a particular type of survivor cells called persisters [3]. Furthermore, the biofilm matrix itself may act as a barrier retarding the diffusion of antibiotics into biofilms as seen in a recent study of tobramycin penetration into non-mucoid *P. aeruginosa* biofilm [2]. Due to these physiological properties of biofilms, distinct from the surrounding tissue during biofilm infections, Cao *et al*. suggested that biofilms with their matrix form a third and independent compartment with exclusive pharmacokinetics important for the effect of antibiotics [4]. If the pharmacokinetics of an antibiotic within the biofilm is very particular, this effect could be substantial even in relatively small biofilms and contribute to the observed reduced bacterial killing inside biofilms.

Positively charged aminoglycosides can bind to the negatively charged exopolysaccharide alginate produced in e.g. *P. aeruginosa* biofilm microcolonies [4–7]. The binding of tobramycin is not expected to change the equilibrium concentration of free antibiotics inside the biofilm [8]. However, it has been speculated that the binding could produce sufficient retardation of the exposure to allow for cells in the biofilm to adapt to the antimicrobial agent [2, 9, 10]. With the binding-induced delayed diffusion, in combination with a relatively short half life reducing the surrounding concentrations of tobramycin, there is a risk that the central zones of biofilm colonies will not experience sufficient exposure of free tobramycin for bacterial killing. In order to investigate this effect, we have measured the binding of tobramycin as a function of external tobramycin concentration in the seaweed alginate biofilm matrix model recently presented by Christophersen, Cao and coworkers [4, 11]. The findings from these experiments are presented here.

## Materials and Methods

Seaweed alginate beads were prepared by modified procedure from that previously described by Cao *et al*. [4, 11]. A syringe containing 3% alginate (Protanal LF 10/60, FMC BioPolymer, Drammen, Norway) was placed in a syringe pump and beads were generated at an alginate flow rate of 40 ml/h. The syringe was connected to either a plastic tube or a needle to create beads in various sizes. The tip of the plastic tube/needle was fixed exactly 8 cm above the surface of a gelling bath of Tris-HCL with CaCl_2_, that was placed on a magnetic stirrer to prevent the beads from adhering. The beads were kept in the gelling bath for at least one hour to stabilise and harden. Afterwards the beads were washed twice with 0.9% NaCl with 12.5 mM CaCl_2_ as a stabiliser. The antibiotic tobramycin sulphate (ApodanNordic, Copenhagen, Denmark) was used in the experiments.

**Fig 1:**
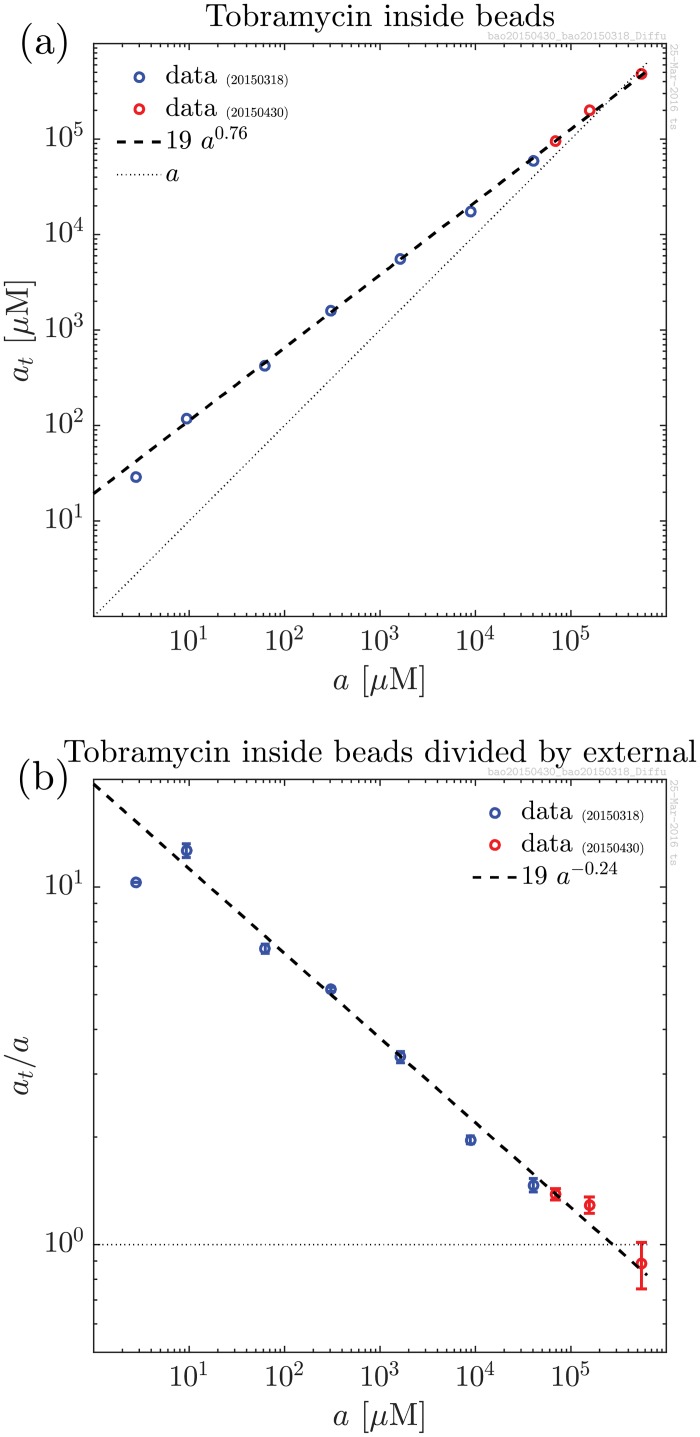
Equilibrium concentration of Tobramycin. a) Total concentration, *a*_*t*_, of tobramycin inside alginate beads as a function of external tobramycin concentration. The total concentration, i. e. free + bound, follows a power law over 5 decades covering from well below typical clinically relevant concentrations to well above. Red and blue data points were obtained in independent experiments. b) Normalised concentration, *a*_*t*_/*a*, of tobramycin inside alginate beads as a function of external tobramycin concentration.

In one series of experiments, a known total volume, *V*_*b*_, of beads is added to a buffered solution of antibiotics at concentration *a*_0_. After equilibration, the concentration of antibiotics in the solution, *a*, was measured using a Thermo Scientific Indiko Clinical and Specialty Chemistry System (Thermo Fisher Scientific, Waltham, Massachusetts, USA). Average values after 8 h, 16 h, and 24 h equilibration were used. As this equipment is designed for clinical use, the lower and upper measurement limitations for tobramycin concentrations are 0.5–10 mg/l, respectively. (Tobramycin has molar mass 467.5g/mol, i. e. 1 mg/l corresponds to 2.14 *μ*M.) Measurements beyond this interval were diluted manually with 0.9% NaCl before analysing. Since we deviate from the manufacturers instructions by working in a solution without serum, the dilution and measurement processes were checked in the range of our measurement and the accuracy of the measurement was confirmed (S1 Fig). The total concentration inside the beads is calculated as *a*_*t*_ = (*V*_*e*_/*V*_*b*_)(*a*_0_ − *a*). These experiments were performed with 20 beads with total volume *V*_*b*_ = 1 ml in each measurement (radius approximately 2.3 mm). The external volume was *V*_*e*_ = 2 ml with concentrations of tobramycin in the range 10^0^–10^5^ mg/l. Resulting total concentrations in the beads as a function of external concentration are shown in Fig 1a. The resulting curve follows a power law with power 0.76. In Fig 1b the same data are shown normalised to the external concentration.

**Fig 2:**
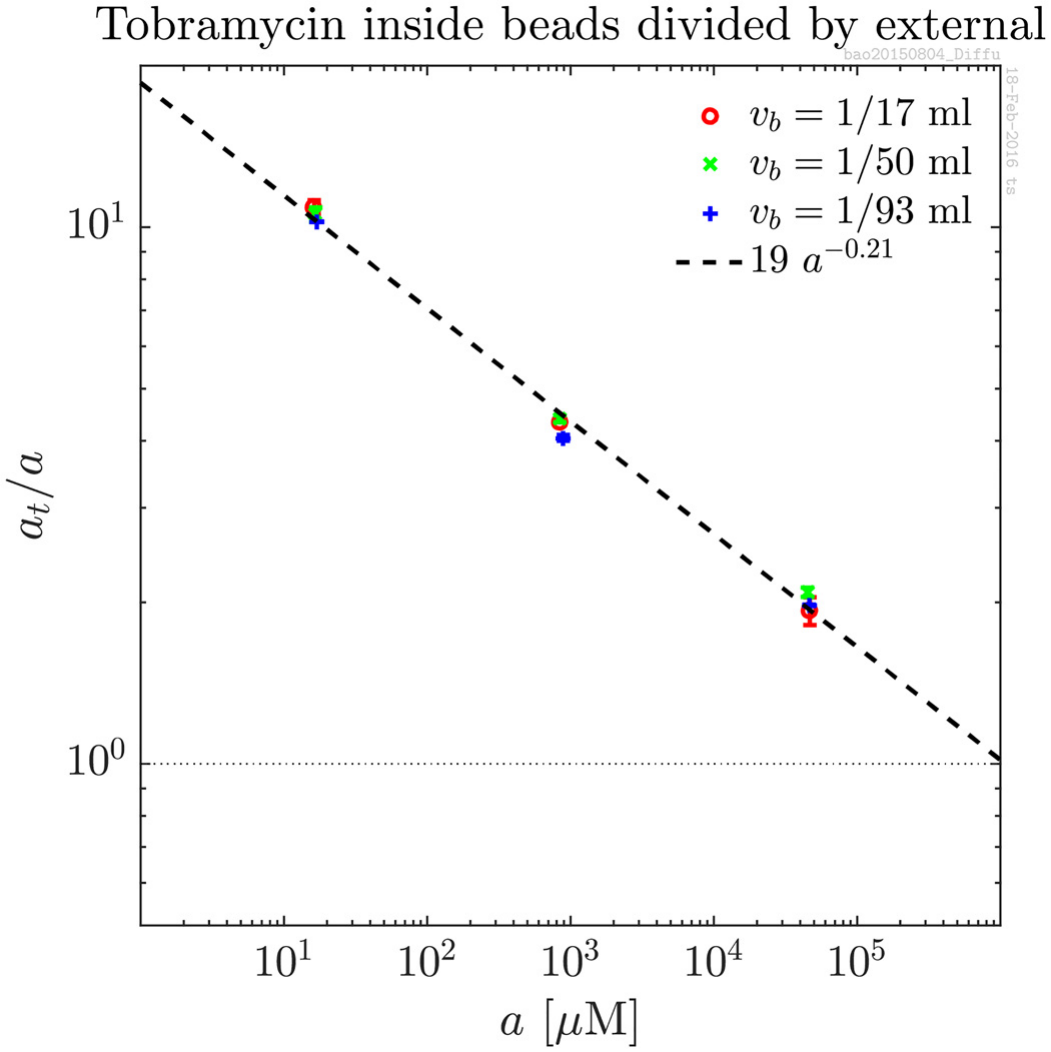
Equilibrium concentrations of tobramycin inside alginate beads normalised to external for three different volumes of individual beads: 1/17 ml, 1/50 ml, and 1/93 ml. The total volume of the beads is kept constant at 1 ml by using 17, 50, and 93 beads were used in the three data sets respectively. The surface/volume ratio is thereby varied a factor 1.8 without changing the total volume of the beads. The data is consistent with homogeneous distribution of tobramycin in the beads.

In order to verify that the tobramycin is indeed distributed homogeneously in the beads, a series of experiments was performed using different sized beads thereby varying the surface/volume ratio by a factor 1.8. The total volume of the beads was kept constant at 1 ml while doing so. Specifically, 17 beads of volume 1/17 ml each, 50 beads of volume 1/50ml, and 93 beads of volume 1/93 were used. The results are shown in Fig 2. The tobramycin accumulation is seen to be independent of the volume of the individual beads and thereby independent of the surface/volume ratio. This is consistent with homogeneous distribution of the tobramycin in the beads.

This experiment was performed using a new batch of alginate and is therefore also an independent control of the first experiment. The deduced power law behaviour is consistent in the two experiments.

## Analysis

The analysis is split into three subsections. In “Reaction-diffusion model” we describe a conventional reaction-diffusion model of the tobramycin binding. In “Nonspecific binding” we bring the observed power law in place and argue that it may be seen as a result of binding to different sites in the alginate with different affinities. Finally, in “Reaction-diffusion model with power-law binding” we present a simplified reaction-diffusion model and the numerical solution of the model.

### Reaction-diffusion model

As a first attempt to understand the influence of the biofilm on diffusion properties of tobramycin, let us consider first order binding of tobramycin to a single binding site in the biofilm matrix. The reaction-diffusion equations are then

$$\begin{array}{c} \frac{\partial a}{\partial t}=D\Delta a-k^{+}a\;m_{f}+k^{-}m_{b}-\lambda \;a \end{array}$$ (1)

$$\begin{array}{c} \frac{\partial m_{b}}{\partial t}=k^{+}a\;m_{f}-k^{-}m_{b} \end{array}$$ (2)

$$\begin{array}{c} m_{t}=m_{f}+m_{b} \end{array}$$ (3)

where *a* is the molar concentration of free antibiotics, i. e. antibiotics that is not bound to the matrix. The first term in Eq (1) describes the isotropic diffusion of the unbound antibiotics with diffusion constant *D*. The next two terms account for association of the antibiotics to free matrix binding sites for the antibiotics with molar concentration *m*_*f*_ and dissociation of antibiotics from occupied sites with molar concentration *m*_*b*_. The corresponding rate constants are denoted *k*^+^ and *k*^−^. The last term is a hypothetical consumption/degradation of the free antibiotics with rate *λ*, e. g. representing defence from the bacteria. The free and occupied binding sites add up to the total Eq (3). Volume exclusion and obstruction effects may be assumed small at low concentration of polymeric substance [12–15].

In the limit of slow degradation and diffusion, quasistatic binding to the matrix can be assumed, i. e. Eq (2) may be set equal to zero. This leads to a total concentration of antibiotics inside the biofilm

$$\begin{array}{c} a_{t}=a+m_{b}=a+\frac{a}{K+a}\;m_{t} \end{array}$$ (4)

where *K* = *k*^−^/*k*^+^ is the dissociation constant for the binding of antibiotics to the biofilm matrix. Saturation sets in when *a* ∼ *K* and full saturation at *a* ∼ (*K* + *m*_*t*_). While this is very different from the observed power-law behaviour for tobramycin binding in Fig 1a, we shall proceed applying it in the most basic model. Below, in “Nonspecific binding”, we shall see how the first order binding connects to the power-law behaviour.

The total antibiotics economy evolves relatively slowly and can be isolated by adding Eqs (1) and (2)

$$\begin{array}{c} \frac{\partial a_{t}}{\partial t}=D\Delta a-\lambda a \end{array}$$ (5)

The right-hand side reflects that only the free antibiotics participates in the diffusion (or is consumed). By insertion of Eq (4) in Eq (5) we finally arrive at

$$\begin{array}{c} \left(\;1+\frac{K^{2}}{{\left(K+a\right)}^{2}}\;\frac{m_{t}}{K}\;\right)\;\;\frac{\partial a}{\partial t}=D\Delta a-\lambda a \end{array}$$ (6)

as a reasonably simple expression which describes the free antibiotics household.

The effect of the binding of the antibiotics to the biofilm matrix is therefore to stretch the time needed to penetrate into the biofilm by a factor

$$\begin{array}{c} “\text{time scaling}”=1+\frac{K^{2}}{{\left(K+a\right)}^{2}}\;\frac{m_{t}}{K} \end{array}$$ (7)

which remains a function of space and time via the antibiotics concentration, *a*. If the concentration of binding sites is low there will be no effect of storing antibiotics in the matrix. Similarly, if the free antibiotics concentration is high, all sites will quickly be occupied, and the effect of the storage on the timing will be reduced.

### Nonspecific binding

**Fig 3:**
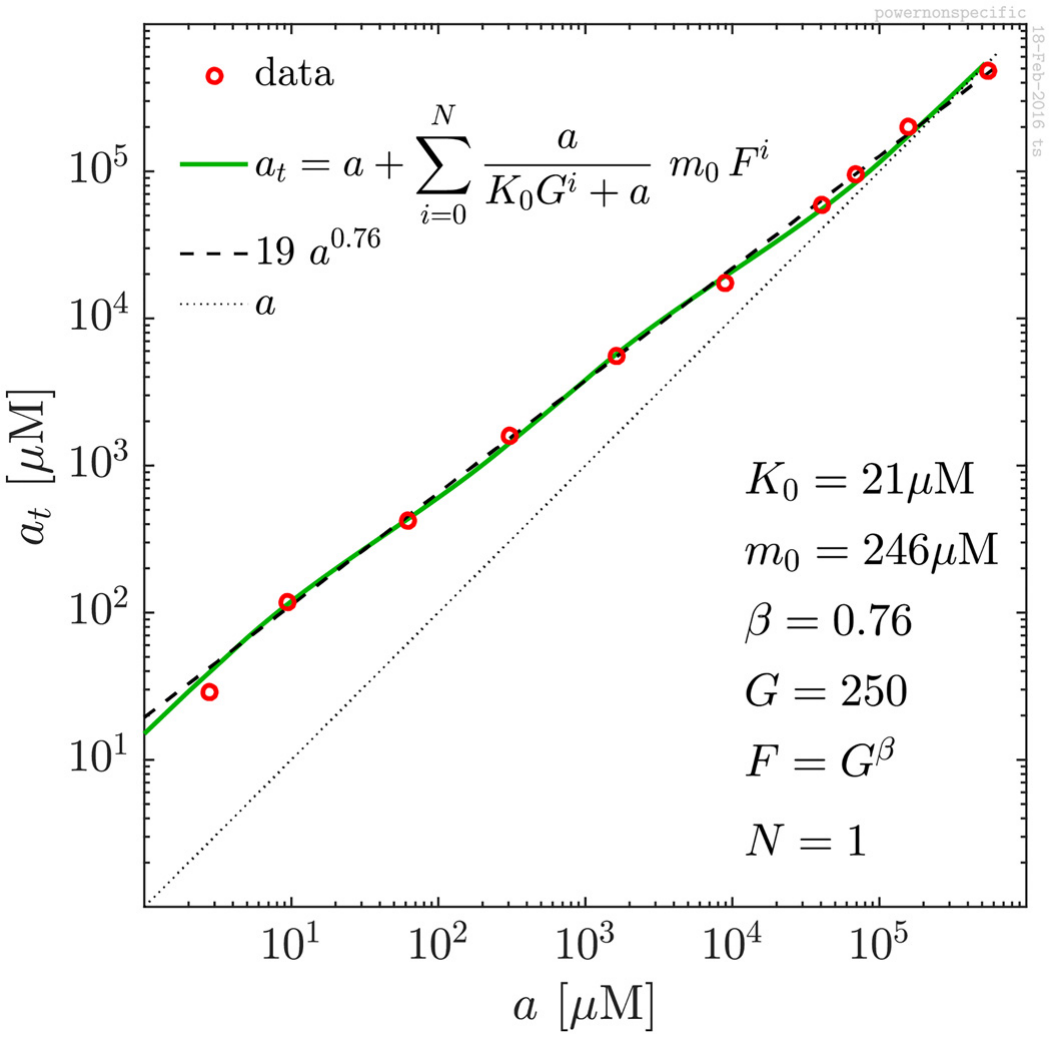
Nonspecific binding model. Example of a fit of the observed power law using the simple nonspecific binding model outlined in the text. The parameters are specified in the figure.

The positively charged tobramycin generally binds well to the polysaccharide alginate matrix [5–7]. Given the variation in the composition and size of the polysaccharide, a plausible origin for the observed power-law behaviour would be binding to a number of different binding sites in the alginate. Binding sites with higher dissociation constants correspond to higher off-rates, which, in turn, occur with higher probability. As an example, a sum of contributions from bindings to sites with dissociation constants *K*_0_ *G*^*i*^ and corresponding concentrations *m*_0_*F*^*i*^, *i* = 0, …, *N*,

$$\begin{array}{c} a_{t}=a+\sum_{i=0}^{N}m_{0}\;F^{i}\;\frac{a}{K_{0}G^{i}+a} \end{array}$$ (8)

can reproduce the observed power-law behaviour for a range of choices of the parameters, *K*_0_, *G*, *m*_0_, *F*, and *N*. This is just a weighted sum of contributions of the form in Eq (4). We find that the *F*^*i*^ = *G*^*βi*^ in the weights ensures that it adds up to a power law with power around *β*. For large *N* and below saturation this approximation may be deduced by induction. We expected that many terms would be needed to produce a power law over five decades, and that it would therefore be hard to argue that this explanation were valid. However, in Fig 3 we show that a fit with just two terms in the sum suffices. (*N* = 1, *β* = 0.76, *K*_0_ = 21*μ*M, *m*_0_ = 246*μ*M, and *G* = 250.) More terms in the sum improves but slightly the fit. Bearing in mind the polyanion nature of the alginate, such a nonspecific binding model for tobramycin to the matrix appears reasonable. (The specific fit corresponds to a systematic change in number of non-specific sites, *F* = exp(*βE*/*k*_*B*_ *T*), as function of the binding energy, *E*, where *k*_*B*_ is the Boltzmann constant and *T* the absolute temperature [16]. The exponent of *β* ∼ 0.76 corresponds to 66 times more non-specific sites with 3.3 kcal/mol weaker binding. The scaling over a factor 10^5^ seen in Fig 1 suggests this relation to hold over an energy range of about 10kcal/mol.)

### Reaction-diffusion model with power-law binding

Let us now include the observed power-law

$$\begin{array}{c} a_{t}=\alpha \;a^{\beta } \end{array}$$ (9)

which describes the quasi-static binding of tobramycin to the alginate matrix in place of Eq (4) in the reaction-diffusion model. Insertion into Eq (5) leads to

$$\begin{array}{c} \alpha \beta \;a^{\beta -1}\;\frac{\partial a}{\partial t}=D\Delta a-\lambda a \end{array}$$ (10)

as the equation describing the tobramycin penetration. Thus, the binding of the antibiotics to the biofilm matrix stretches the time needed to penetrate into the biofilm by a factor

$$\begin{array}{c} “\text{time scaling}”=\alpha \beta \;a^{\beta -1} \end{array}$$ (11)

which remains a function of space and time through the antibiotics concentration, *a*.

### Numerical solution

**Fig 4:**
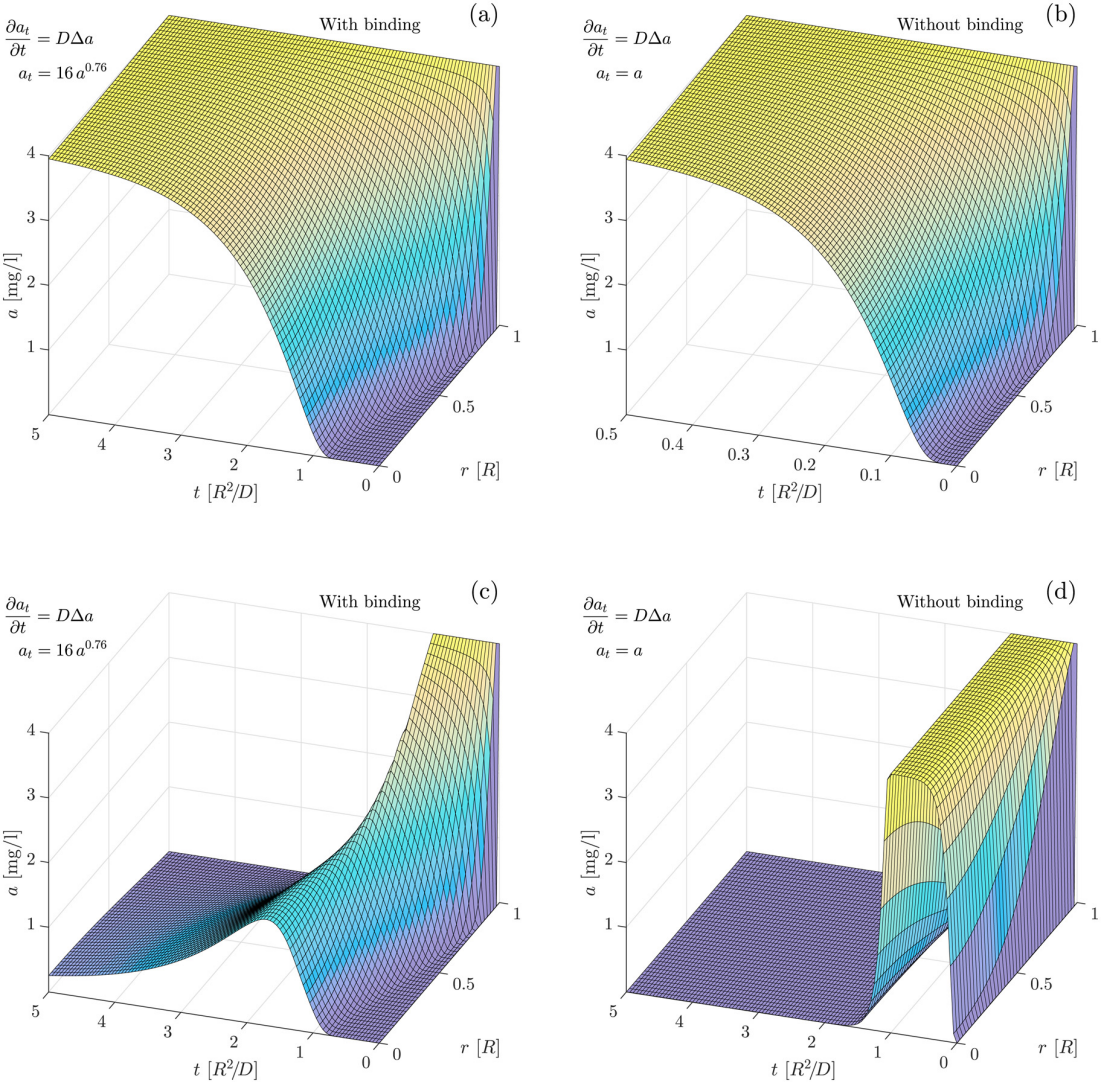
Tobramycin diffusion. Numerical solution of the reaction-diffusion equation with power-law binding to the biofilm matrix in a spherical bead with radius *R* and diffusion constant *D* for the free tobramycin. The free tobramycin is displayed as a function of radius and time. Time is expressed in units of *R*^2^/*D*. a) With binding. The external concentration of tobramycin is kept at *a* = 4 mg/l from time *t* = 0. The maximum time shown is *t* = 5*R*^2^/*D*. b) Without binding. The maximum time shown is *t* = 0.5*R*^2^/*D*. c) With binding, external concentration is kept at *a* = 4 mg/l from time *t* = 0 to *t* = *R*^2^/*D*. d) Without binding, external concentration is kept at *a* = 4 mg/l from time *t* = 0 to *t* = *R*^2^/*D*.

Numerical solutions of the reaction-diffusion equation with power-law binding, i.e. Eq (10), are shown in Fig 4. The numerical integration has been performed using the numerical solver by Skeel *et al*. [17, 18] with adaptive discretisation set to meet the relative tolerance 10^−3^ and the absolute tolerance 10^−6^. Free antibiotics is introduced at concentration of *a* = 4 mg/l at the surface of the bead at time *t* = 0. This is a bit above the peak concentration in sputum of CF patients during intravenous treatment [19, 20]. Fig 4a shows how the antibiotics diffuses into the bead when binding is included. We observe full penetration only after *t* ∼ 5*R*^2^/*D*. In Fig 4b the binding is turned off and we observe full penetration already at *t* ∼ 0.5*R*^2^/*D*, i. e. an order of magnitude faster. Qualitatively, this is in accordance with the filtering predicted by Eq (11).

Fig 4c and 4d show how a transient introduction of antibiotics at the surface is low-pass filtered when it reaches the center of the biofilm bead. When we allow for binding of the tobramycin to the biofilm matrix, the concentration of free antibiotics at the center is lowered but sustained for a longer time.

## Results and Discussion

The stationary concentration of tobramycin inside alginate beads was measured as a function of the external concentration. As can be seen in Fig 1a, it follows a power law to a good approximation. In typical units used in medicine or molecular biology the observed power law reads

$$\begin{array}{c} a_{t}=19\;\mu M\;{\left(\;\frac{a}{1\;\mu M}\;\right)}^{0.76} \end{array}$$ (12)

$$\begin{array}{c} a_{t}=16\;\text{mg}/l\;{\left(\;\frac{a}{1\;\text{mg}/l}\;\right)}^{0.76} \end{array}$$ (13)

The accumulation of tobramycin was confirmed to be consistent with homogeneous distribution in the beads. This was done in a series of experiments where the surface-to-volume ratio was varied a factor two without changing *a*_*t*_/*a* as shown in Fig 2. The power law was found to be consistent with nonspecific binding to sites in the polyanion biofilm matrix.

In Fig 1b we observe that the ratio, *a*_*t*_/*a*, of concentrations of tobramycin inside the beads to the external concentration keeps rising as the tobramycin concentration goes down. The effective diffusion constant, *D*/(*αβa*^*β*−1^), is proportional to (*a*_*t*_/*a*)^−1^. Therefore the diffusion becomes slower at low concentrations of tobramycin where the non-specific binding of antibiotics molecules is relatively stronger. This explains why, in the work of Cao *et al*. [4], it was observed that it is very hard to “wash out” the tobramycin from the beads.

The power-law scaling of the binding also implies that, when tobramycin is introduced at the surface of the bead, a “front” builds up as the low number of antibiotics molecules ahead of the front binds more and thereby moves slower than the bulk of the diffusing molecules. The front is seen quite clearly in Fig 4a and 4c.

At typical clinical doses, ∼5 mg/kg, the peak serum concentration is of order ∼20 mg/kg [19], and the peak concentration in sputum is ∼3.6 mg/l during intravenous treatment [20]. At this concentration, the time stretching is about a factor 9 growing to a factor 12 at *a* ∼ 1 mg/l. The diffusion constant for tobramycin in water at 37°C is around *D* = 1.4 mm^2^/h [7]. For a medium sized biofilm bead of radius *R* = 100 *μ*m [9, 21–25] the characteristic time, *R*^2^/*D*, is 0.4 min which, by the time scaling, becomes about 4 min. In the human body, the elimination half life for an aminoglycoside (gentamicin) is of order 2.0 h [26], i. e. much slower than the diffusion time. In order to aid in protecting the bacteria against tobramycin treatment, the tobramycin buffering would therefore have to act in concert with other mechanisms, e. g. an active defence mechanism of the bacteria.

However, for a very large sized microcolony, say *R* ∼ 1 mm, the filtering is, but marginally, able to keep the concentration of free tobramycin at the center of the biofilm below the MIC of 2 mg/l during treatment. Microcolonies this large have only been reported on foreign bodies [24] and not in sputum of CF patients.

In the present study, we have kept the composition of the biofilm matrix constant with a concentration of 3%. Clinical extracts display alginate concentrations ranging from 0.4% to 10% with a median around 3.5% [27, 28]. We expect that the prefactor, *α*, will be proportional to the density of the biofilm matrix. Thus the low-pass filtering will be even more pronounced in the more dense biofilm aggregates and will likely play an important role in the protection of the bacteria.

## Conclusions

The penetration and binding of tobramycin into seaweed alginate beads have been measured. The tobramycin is found to be homogeneously distributed in the beads. Surprisingly, we find that the total concentration of tobramycin inside the biofilm matrix displays a power-law dependence on the external concentration over five decades. Non-specific binding of tobramycin to the polyanion polysaccharide biofilm matrix was presented as a plausible explanation for the observed power-law.

The observed power-law behaviour was used in a reaction-diffusion model to demonstrate that the tobramycin storage capacity is sufficient to produce pronounced low-pass filtering of the free tobramycin concentration inside the bead. These calculations also showed that in very large colonies the delay caused by the binding is sufficient to keep the tobramycin below the MIC level at the center.

In clinical extracts, biofilm aggregates display algenate densities ranging from 0.4% to 10%. In the more dense microcolonies of this distribution, we expect the retardation of the diffusion to contribute significantly to the protection of bacteria residing in the biofilm.

## Supporting Information

S1 Fig Check of calibration. The concentration of tobramycin in the buffer, *a*, measured as described in versus the true concentration, *a*_0_.
(PDF)

S1 Data Data underlying Figs 1 and 2. Equilibrated concentrations for the two series of experiments described in Materials and Methods.
(PDF)
