# Supplementary figures and images for: Diffusion Retardation by Binding of Tobramycin in an Alginate Biofilm Model
Source: PLoS One. 2016 Apr 21;11(4):e0153616. doi: 10.1371/journal.pone.0153616 (PMC4839563; doi:10.1371/journal.pone.0153616)

# Check of calibration

bao20150430\_bao20150318\_Diffu

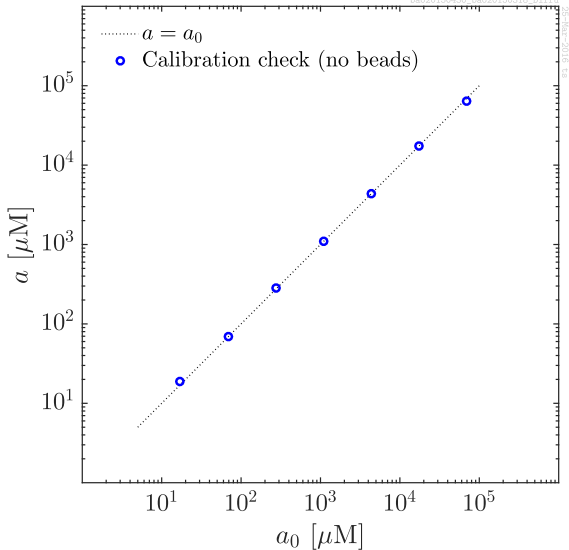

25-Mar-2016 ts

Supplement: S1 Fig — The concentration of tobramycin in the buffer, a, measured as described in versus the true concentration, a0. (PDF) [file pone.0153616.s001.pdf]
