# Supplementary material for: Diffusion Retardation by Binding of Tobramycin in an Alginate Biofilm Model
Source: PLoS One. 2016 Apr 21;11(4):e0153616. doi: 10.1371/journal.pone.0153616 (PMC4839563; doi:10.1371/journal.pone.0153616)
Supplement: S1 Data — Equilibrated concentrations for the two series of experiments described in Materials and Methods. (PDF) [file pone.0153616.s002.pdf]

DATA USED IN Fig. 1a AND 1b

First column is the initial concentration of tobramycin in the buffer. The next three colums is the concentration after 8, 12, and 24 hours of equilibration.

The ”a” is the averaged, and at is the calculated concentration inside the beads. Color code as in Fig. 1

2015.03.18

a0 [mg/l]\ time [	8.00	12.00	24.00	**a (x-axis)**	**at (y-axis, 1a)**	**at/a (y-axis, 1b)**
8.00	1.30	1.30	1.30	**1.30**	**13.40**	**10.31**
32.00	4.20	4.70	4.20	**4.37**	**55.27**	**12.66**
128.00	28.00	30.00	30.00	**29.33**	**197.33**	**6.73**
512.00	140.00	144.00	144.00	**142.67**	**738.67**	**5.18**
2048.00	735.00	768.00	795.00	**766.00**	**2564.00**	**3.35**
8192.00	4080.00	4080.00	4240.00	**4133.33**	**8117.33**	**1.96**
32768.00	18300.00	19500.00	18900.00	**18900.00**	**27736.00**	**1.47**

2015.04.30

a0 [mg/l]\ time [	6.00	13.25	24.00			
54,600.00	33,200.00	31,600.00	32,000.00	**32,266.67**	**44,666.67**	**1.38**
120,000.00	70,000.00	75,000.00	73,750.00	**72,916.67**	**94,166.67**	**1.29**
370,000.00	233,331.00	269,997.00	266,664.00	**256,664.00**	**226,672.00**	**0.88**

DATA USED IN Fig. 2

Concentration of tobramycin for three sizes of beads as a function of time. Only the saturated values are used. Color coded are the data plotted in Fig 2.

The two last equilibrated data points (bold) are considered equilibrated and used in each averaging.

Bead\time [h]	a0	12.00	16.00	24.00	**a (x-axis)**	at (2/1*(a0-a))	**at/a (y-axis)**
17 x 1/17ml	**49.00**	9.00	**7.80**	**7.40**	7.60	82.80	**10.89**
17 x 1/17ml	**1,260.00**	475.00	**398.00**	**398.00**	**398.00**	1,724.00	**4.33**
17 x 1/17ml	**43,000.00**	23,000.00	**21,250.00**	**22,525.00**	**21,887.50**	42,225.00	**1.93**
50 x 1/50ml	**49.00**	8.70	**7.60**	**8.00**	**7.80**	82.40	**10.56**
50 x 1/50ml	**1,260.00**	450.00	**390.00**	**398.00**	**394.00**	1,732.00	**4.40**
50 x 1/50ml	**43,000.00**	23,850.00	**20,825.00**	**21,250.00**	**21,037.50**	43,925.00	**2.09**
93 x 1/93ml	**49.00**	9.30	**8.00**	**8.00**	**8.00**	82.00	**10.25**
93 x 1/93ml	**1,260.00**	400.00	**413.00**	**420.00**	**416.50**	1,687.00	**4.05**
93 x 1/93ml	**43,000.00**	**21,600.00**	**21,675.00**	N/A	**21,637.50**	42,725.00	**1.97**
